# Thomas A. Means, M. D.

**Published:** 1859-05

**Authors:** 


THOS. A. MEANS, M. D.

We would direct special attention to an interesting article upon Spermatorrhœa, in this number of our Journal, from our friend and former pupil, Dr. Thos. A. Means, of Memphis, Tennessee.

After pursuing the study of his profession under the direction and instruction of his distinguished father, Prof'r Alex'r Means, M.D., long Prof. of Chemistry in the Medical College of Georgia, at Augusta, and President of Emory College, Oxford, and now Prof. of Chemistry and Pharmacy in the Atlanta Medical College, and availing himself of the advantages of American Medical Colleges, and the degree of M.D., he repaired to Paris, where he spent a considerable time in industrious application to the acquisition of a knowledge of his profession, and has now settled in Memphis, where we predict for him a most successful career. We hope to be able to present to our readers frequent contributions from his pen.

